# The Evolving Role of Hematopoietic Stem Cell Transplantation in Philadelphia-like Acute Lymphoblastic Leukemia: From High-Risk Standard to Precision Strategies

**DOI:** 10.3390/cancers17193237

**Published:** 2025-10-05

**Authors:** Matteo Molica, Claudia Simio, Laura De Fazio, Caterina Alati, Marco Rossi, Massimo Martino

**Affiliations:** 1 Department of Hematology-Oncology, Azienda Universitaria Ospedaliera Renato Dulbecco, 88100 Catanzaro, Italy; claudia.simio@studenti.univr.it (C.S.); laura.defazio@aourenatodulbecco.it (L.D.F.); rossim@unicz.it (M.R.); 2 Hematology and Stem Cell Transplantation and Cellular Rerapies Unit (CTMO), Department of Hemato-Oncology and Radiotherapy, Grande Ospedale Metropolitano “Bianchi-Melacrino-Morelli”, Presidio Morelli, 89128 Reggio Calabria, Italy; caterina.alati@ospedalerc.it (C.A.); massimo.martino@ospedalerc.it (M.M.)

**Keywords:** Philadelphia-like ALL, hematopoietic stem cell transplantation, measurable residual disease, tyrosine kinase inhibitors, JAK-STAT pathway, genomic profiling, CAR-T cell therapy, high-risk leukemia

## Abstract

**Simple Summary:**

Philadelphia-like acute lymphoblastic leukemia (Ph-like ALL) is a high-risk form of B-cell ALL, characterized by kinase-activating genetic alterations and a poor response to conventional chemotherapy. This review analyzes the role of allogeneic hematopoietic stem cell transplantation (allo-HSCT), highlighting that transplantation in first remission may improve survival in patients with high molecular risk or positive measurable residual disease (MRD). Personalized therapeutic approaches based on genomic profiling and MRD assessment are becoming essential. New strategies, such as immunotherapy, TKIs, JAK inhibitors, and CAR-T cells, may improve clinical outcomes and, in some cases, prevent the need for allo-HSCT.

**Abstract:**

Background: Philadelphia-like acute lymphoblastic leukemia (Ph-like ALL) is a high-risk subtype of B-cell ALL characterized by a gene expression profile similar to BCR::ABL1-positive leukemia, but lacking the BCR::ABL1 fusion gene. It is frequently associated with kinase-activating alterations, such as CRLF2 rearrangements, JAK-STAT pathway mutations, and ABL-class fusions. Patients with Ph-like ALL typically experience poor outcomes with conventional chemotherapy, underscoring the need for intensified and targeted therapeutic approaches. Methods: This review summarizes current evidence regarding the role of hematopoietic stem cell transplantation (HSCT) in patients with Ph-like ALL. We analyzed retrospective cohort studies, registry data, and ongoing clinical trials, focusing on transplant indications, molecular risk stratification, measurable residual disease (MRD) status, timing of transplant, and post-transplant strategies. Results: Retrospective data suggest that HSCT in first complete remission (CR1) may improve survival in patients with high-risk molecular lesions or MRD positivity at the end of induction. However, the lack of prospective data specific to Ph-like ALL limits definitive conclusions. Post-transplant relapse remains a challenge, and novel strategies, including the use of tyrosine kinase inhibitors or JAK inhibitors as post-HSCT maintenance therapy, are being explored. Emerging immunotherapies, such as chimeric antigen receptor (CAR) T cells, may reshape the therapeutic landscape and potentially alter the indications for transplantation. Conclusions: HSCT remains a crucial therapeutic option for selected patients with Ph-like ALL, particularly those with poor molecular risk features or persistent MRD. However, further prospective studies are needed to evaluate the indication for HSCT in CR1 and the potential integration of transplantation with targeted and immunotherapeutic strategies. Personalized treatment approaches based on genomic profiling and MRD assessment are essential to improve outcomes in this high-risk subset.

## 1. Introduction

Philadelphia-like acute lymphoblastic leukemia (Ph-like ALL), also referred to as BCR::ABL1-like ALL, represents a recently characterized high-risk subtype of B-cell precursor acute lymphoblastic leukemia (B-ALL). Initially identified and described in 2009 through gene expression profiling studies, Ph-like ALL exhibits a transcriptional signature akin to BCR::ABL1-positive ALL but lacks the BCR::ABL1 fusion gene. The entity described was initially recognized as a provisional subtype of B-cell acute lymphoblastic leukemia (B-ALL) in the 2016 WHO classification and later elevated to a full subtype in the 2022 WHO and International Consensus Classification (ICC) of lymphoid neoplasm [1]. The ICC classification categorizes Ph-like ALL into three classes: ABL-rearranged, JAK-STAT activated, and not otherwise specified (NOS) [2,3,4,5]. Its prevalence varies by ethnicity (more common in Hispanic populations) and by age, accounting for 10–15% of B-ALL cases in children and 20–30% in adults, with a peak in young adults [6,7].

Its molecular heterogeneity and frequent association with high white blood cell counts, poor early treatment response, and elevated rates of measurable residual disease (MRD) contribute to an overall poor prognosis under conventional chemotherapy regimens [3,8,9,10,11,12,13,14,15,16,17,18]. Although the application of pediatric-inspired intensive regimens has improved outcomes in adolescents and young adults (AYA) patients, relapse rates remain unacceptably high in the Ph-like subset, necessitating the exploration of risk-adapted, genomically informed therapeutic strategies [11].

Hematopoietic stem cell transplantation (HSCT) has long been established as a consolidation strategy for patients with high-risk ALL in first complete remission (CR1) or relapsed/refractory disease, particularly when MRD persists after induction or consolidation [13,14]. Given the dismal outcomes of Ph-like ALL under standard approaches, HSCT is frequently considered in this population. However, the precise indications for transplant in the context of Ph-like ALL remain poorly defined due to the paucity of prospective data and the wide variability in molecular lesions [15]. Furthermore, the increasing availability of targeted therapies, such as tyrosine kinase inhibitors (TKIs) and JAK inhibitors, introduces novel therapeutic options that may affect both the timing and the necessity of HSCT [3,16,17].

This review aims to provide a comprehensive overview of the current evidence regarding the role of HSCT in Ph-like ALL. We summarize the molecular landscape and clinical characteristics of this entity, analyze transplant-related outcomes from retrospective and prospective studies, and discuss the impact of MRD and genomic risk stratification on transplant decisions. Finally, we explore emerging treatment paradigms, including post-transplant maintenance therapy and the evolving role of cellular immunotherapies, which may reshape the future of Ph-like ALL management.

## 2. Biological Landscape of Ph-like ALL

Ph-like ALL is defined by a gene expression profile resembling that of BCR::ABL1-positive ALL, but without the presence of the BCR::ABL1 fusion gene. Instead, it comprises a molecularly heterogeneous group of leukemia unified by the activation of cytokine receptor and kinase signaling pathways, many of which are potentially targetable. These lesions contribute to leukemogenesis by promoting aberrant cell proliferation and survival and are associated with poor treatment response and increased relapse risk [3,7]. A large multicenter study by Roberts et al., observed poorer outcomes for patients with Ph-like acute lymphoblastic leukemia compared to those with non–Ph-like acute lymphoblastic leukemia among young adults and adults, with 5-year event-free survival rates of 24.1%. Specifically, patients with CRLF2 or JAK2/EPOR rearrangements showed a trend toward lower 5-year event-free and overall survival compared to patients with ABL-class alterations or other JAK-STAT pathway alterations [9].

### Major Genomic Subtypes and Diagnostic Approaches

The majority of Ph-like ALL cases harbor fusion genes involving tyrosine kinases or rearrangements of cytokine receptors, often accompanied by mutations in components of the JAK/STAT signaling pathway. Based on these genetic alterations, Ph-like ALL can be classified into distinct molecular subgroups, including:

- CRLF2 rearrangements (approximately 50% of pediatric and AYA Ph-like cases). These include translocations of CRLF2 to the IGH locus (IGH::CRLF2) or interstitial deletions of the pseudoautosomal region 1 (PAR1), leading to P2RY8-CRLF2 fusions. These alterations often co-occur with activating mutations in JAK1 or JAK2, resulting in constitutive JAK-STAT signaling [4,6].
  Patients with CRLF2 rearrangement and concomitant JAK2 mutation tend to have significantly worse outcomes compared to patients with CRLF2 rearrangement but JAK2 wild-type, with 5-year survival rates below 20% [15].
- ABL-class fusions (10–15%). These include rearrangements involving ABL1, ABL2, CSF1R, or PDGFRB, which are functionally similar to the BCR::ABL1 fusion and responsive to ABL tyrosine kinase inhibitors such as imatinib or dasatinib [9,10]. ABL-class fusions are usually mutually exclusive with CRLF2 rearrangements.
- JAK-STAT and RAS pathway mutations. These include mutations in JAK1, JAK2, IL7R, SH2B3, and FLT3, or in RAS pathway genes such as KRAS, NRAS, and NF1. These mutations may occur independently or in combination with other lesions, and support the rationale for JAK inhibitors or MEK inhibitors in experimental protocols [5,19].

Less commonly, rearrangements involving EPOR, TYK2, NTRK3, and BLNK have been identified, further expanding the kinase-activated landscape of Ph-like ALL [20,21].

The diagnosis of Ph-like ALL remains challenging due to the absence of a single pathognomonic lesion. Current approaches typically employ a multi-step algorithm:

- Gene expression profiling (GEP): the gold standard for initial classification but is not routinely available in all clinical laboratories.
- Targeted RNA sequencing and fusion panels: detect kinase fusions and are increasingly used in clinical practice.
- Cytogenetics and FISH: may reveal known rearrangements, although limited in detecting cryptic or novel fusions.
- Next-generation sequencing (NGS) panels: identify mutations in JAK-STAT or RAS pathway genes and can complement RNA-based approaches [21,22].
- Single-nucleotide polymorphism (SNP) arrays: identify genomic abnormalities specifically targeting genes involved in key signaling pathways and are capable of detecting both copy number alterations and copy-neutral loss of heterozygosity (CN-LOH), thereby providing important insights into genomic profiles. Currently, this technique is not routinely available in all clinical laboratories [22].
  Although it is an innovative technique, it is unable to detect some rearrangements that characterize Ph-like ALL.

According to the NCCN (National Comprehensive Cancer Network, 2025) guidelines [1], the early identification of Ph-like lesions is essential for risk stratification and therapeutic management.

Several studies have demonstrated that Ph-like ALL is an independent poor prognostic factor, particularly in patients who fail to achieve MRD negativity early during treatment. Importantly, the presence of CRLF2 rearrangements, JAK-STAT mutations, or ABL-class fusions has been associated with worse event-free (EFS) and overall survival (OS) [23]. Therefore, prompt diagnosis is crucial; nevertheless, it remains a challenging objective due to the lack of standardized guidelines and the heterogeneity of genetic alterations, which are frequently cryptic and not identifiable by conventional methods. While ABL-class fusions may predict response to TKIs, the optimal integration of these agents into frontline regimens and their impact on long-term outcomes, including the need for transplantation, remain areas of active investigation.

## 3. Current Treatment Strategies in Ph-like ALL

Management of Ph-like ALL presents unique clinical challenges due to its genetic heterogeneity, chemo-resistance, and frequent persistence of MRD. Current treatment strategies largely derive from protocols for high-risk B-ALL but are being increasingly adapted to integrate molecular diagnostics and targeted therapies. As prospective data remain limited, most therapeutic decisions are guided by risk stratification, early treatment response, and, when available, genomic profiling.

- JAK inhibitors (e.g., ruxolitinib) in CRLF2-rearranged or JAK-mutant cases
- MEK inhibitors for RAS-driven subtypes [24]

### 3.1. Conventional Chemotherapy Regimens

The backbone of therapy for Ph-like ALL in both pediatric and adult patients remains multi-agent chemotherapy. Pediatric-inspired regimens, such as those developed by the Children’s Oncology Group (COG), have been adapted for AYA and have shown superior outcomes compared to traditional adult protocols in this population [9,11]. However, patients with Ph-like ALL have consistently shown poorer outcomes compared to other B-ALL subtypes, with higher rates of MRD positivity and relapse despite intensive chemotherapy [3,15]. In the COG AALL1131 trial, Ph-like patients treated with standard risk-adjusted chemotherapy exhibited significantly inferior 5-year EFS compared to non–Ph-like counterparts [23]. These findings have prompted interest in augmenting chemotherapy backbones with molecularly targeted agents.

MRD is a powerful predictor of relapse and is increasingly used to guide post-induction therapy decisions. Ph-like ALL patients are more likely to exhibit MRD ≥ 0.01% at end-of-induction, prompting consideration of treatment intensification or early referral for HSCT [13,14]. In both pediatric and adult settings, persistent MRD after induction or consolidation often qualifies as an indication for HSCT, especially when combined with high-risk cytogenetics or kinase-activating lesions. Thus, MRD monitoring is essential for tailoring therapy in Ph-like ALL.

### 3.2. Targeted Therapies in Frontline Regimens

- CRLF2 rearrangements and JAK mutations: preclinical data and early clinical trials support the use of ruxolitinib, a JAK1/2 inhibitor, in cases harboring JAK-STAT pathway activation [25]. The COG AALL1521 trial (NCT02723994) is currently evaluating ruxolitinib in combination with chemotherapy for newly diagnosed Ph-like ALL.
- RAS pathway mutations: these may be susceptible to MEK inhibitors (e.g., trametinib), although clinical validation is still limited [19].
- The ETV6-NTRK3 fusion constitutes a putative therapeutic target for TRK inhibitors (e.g., larotrectinib); however, further rigorous clinical evaluation is necessary [26].
- The EPOR: IGH fusion has demonstrated preclinical sensitivity to combined treatment with ponatinib and ruxolitinib, warranting additional investigation [27].

## 4. The Role of Hematopoietic Stem Cell Transplantation in Ph-like ALL

HSCT remains a cornerstone in the management of high-risk ALL, particularly in patients with persistent MRD or unfavorable genetic features. In Ph-like ALL, HSCT is frequently considered in first complete remission (CR1) [28]. However, the specific role of transplant in this heterogeneous molecular entity remains a subject of ongoing investigation, with limited prospective data to guide standardized recommendations.

### 4.1. Transplant Indications in Ph-like ALL and Outcomes After HSCT

The guidelines of the European Society for Blood and Marrow Transplantation (EBMT) suggest that HSCT should be considered in Ph-like ALL patients with:

- Persistent MRD ≥ 0.01% after induction or consolidation
- High-risk molecular features (e.g., CRLF2 rearrangements, JAK mutations [9])
- Slow early response or induction failure
- Relapsed/refractory disease

In the PETHEMA LAL2019 protocol, patients who present with positive MRD at the end of induction therapy or who exhibit high-risk cytogenetic-molecular features are considered candidates for HSCT [29]. Also, in pediatric protocols (e.g., COG AALL1131), patients with high MRD or adverse genetics are allocated to intensified therapy or HSCT. Similarly, in adult patients treated on MD Anderson or CALGB regimens, transplant is often pursued in CR1 for those with high-risk features [7,23]. Retrospective studies suggest that HSCT may improve survival in Ph-like ALL, especially when performed in CR1. Roberts et al. reported that in AYA patients with Ph-like ALL, those undergoing transplant in CR1 had superior OS and lower relapse rates compared to non-transplanted counterparts [9]. Similarly, Aldoss et al. demonstrated that in adults, HSCT reduced relapse risk and improved leukemia-free survival [12].

However, transplant-related mortality (TRM) remains a significant concern, particularly in older or medically unfit patients. Outcomes are strongly influenced by:

- Disease status at transplant (CR1 vs. CR2 or active disease)
- MRD status pre-HSCT
- Donor source and conditioning intensity
- Presence of targetable lesions and post-HSCT maintenance

In a recent real-world cohort of adults with Ph-like ALL undergoing HSCT, 3-year OS ranged from 40–60%, with relapse remaining the most common cause of failure [30].

### 4.2. Role of MRD in Transplant Decision-Making and Post-HSCT Maintenance Strategies

MRD status at the time of HSCT is the most robust predictor of post-transplant outcomes. Several studies have shown that MRD-positive patients prior to transplant experience significantly higher relapse rates and inferior OS compared to those who are MRD-negative [13,14]. Thus, achieving MRD negativity before transplant, potentially through intensified chemotherapy or the addition of targeted agents, is a key therapeutic objective.

In Ph-like ALL, where MRD persistence is common, early molecular response assessment can help identify patients who would benefit most from HSCT. Ongoing trials are evaluating whether the use of TKIs or JAK inhibitors during induction may enhance early MRD clearance and reduce the need for transplantation.

Given the high relapse/refractory risk in Ph-like ALL even after allo-HSCT, there is growing interest in post-transplant maintenance with targeted therapies:

- TKIs (e.g., dasatinib, ponatinib) may be continued in patients with ABL-class fusions [30]
- JAK inhibitors (e.g., ruxolitinib) are being studied in CRLF2-rearranged or JAK-mutant disease [25]
- Blinatumomab, a bispecific T-cell engager (BiTE), may be used post-transplant in MRD-positive patients

However, prospective data on post-HSCT maintenance in Ph-like ALL remain limited. The randomized ALLG ALL08 and COG trials are evaluating these strategies, and their results will be critical to defining best practices.

### 4.3. Limitations of Current Evidence and Unmet Needs

Most data on HSCT in Ph-like ALL are retrospective, and molecular classification is often incomplete or unavailable. Furthermore, many studies do not stratify outcomes by specific genomic subgroups, limiting interpretation. There is also a lack of consensus on the optimal timing of HSCT in patients who respond well to induction + targeted therapy.

Key unmet needs include:

- Prospective trials evaluating transplant vs. no transplant in MRD-negative, targetable subgroups and/or specific genetic abnormalities.
- Optimal post-HSCT surveillance and pre-emptive intervention strategies
- Integration of immunotherapy and HSCT in frontline or salvage settings

The advent of chimeric antigen receptor T-cell (CAR-T) therapy, particularly CD19-directed products, has significantly impacted the treatment of relapsed/refractory ALL. In patients with Ph-like ALL who relapse after transplant or fail to achieve remission, CAR-T therapy offers a promising salvage option. Whether CAR-T therapy in MRD-positive CR1 could eventually replace HSCT in selected patients is under investigation. Long-term data and direct comparisons between CAR-T and transplant are needed to define their respective roles.

## 5. Comparative Outcomes: HSCT vs. Non-HSCT Approaches

The decision to proceed with HSCT in patients with Ph-like ALL is influenced by multiple factors, including disease burden, molecular alterations, and measurable residual disease MRD status. However, a central question remains: does HSCT provide superior survival benefits over chemotherapy or targeted therapy alone in this high-risk subgroup?

Although no prospective randomized trials have directly compared transplant versus non-transplant strategies in Ph-like ALL, emerging retrospective data and registry analyses offer important insights. Outcomes appear to be heavily modulated by disease risk at baseline, depth of response, and use of targeted agents (Table 1).

Several ongoing trials are attempting to clarify the comparative role of HSCT versus non-HSCT strategies in Ph-like ALL:

- COG AALL1721(NCT03117751): evaluates ruxolitinib in combination with chemotherapy in newly diagnosed Ph-like ALL.
- ALLG ALL08: evaluates ponatinib-based induction in ABL-class fusion ALL and considers HSCT based on MRD.
- ECOG-ACRIN E1910: investigates blinatumomab in MRD-positive B-ALL, including Ph-like subsets.

These studies may help delineate whether targeted agents can mitigate relapse risk sufficiently to avoid HSCT in selected patients.

However, it is fundamental to underline that, at present, there is no definitive evidence confirming the long-term efficacy of this strategy, making it premature to draw solid conclusions on the impact of TKIs in this population. On the contrary, some evidence suggests the need for a more cautious approach. A significant example is represented by the subgroup analysis presented by Dinner et al. at EHA 2025 [23], which highlighted very promising results in young adults with Ph-like ALL treated in the E1910 study, where the addition of blinatumomab led to a significant improvement in survival, without the need to systematically resort to HSCT.

**Table 1:** Ph-like ALL—Comparison of Allogeneic HSCT vs. Non-HSCT Strategies.

	HSCT (Allogenic Stem Cell Transplantation)	No-HSCT (Chemotherapy and/or Target Therapy)
Indication	High-risk molecular profile, MRD positivity after induction	Lower-risk patients or those achieving deep molecular remission
Survival benefit	Retrospective data suggest improved survival in high-risk/MRD+ Patients	Some patients achieve durable remission without HSCT
Toxicity and complications	Higher risk of transplant-related morbidity and mortality	Lower acute toxicity but risk of relapse remains
Role of target agents	Often combined post-HSCT as maintenance	Used as upfront or consolidation therapy; may reduce the need for HSCT
Immunotherapy	Increasingly used post-HSCT or as alternative in some cases	Emerging role as frontline or salvage therapy, potentially avoiding HSCT
Evidence level	Limited prospective trials; mainly retrospective and registry data	Clinical trials ongoing lack of definitive randomized comparison
Ongoing trials addressing	Evaluating HSCT benefit in combination with targeted therapies	Assessing ability of target/immunotherapies to replace or delay HSCT

### 5.1. Retrospective Data Supporting HSCT

Several retrospective studies suggest that HSCT in CR1 may confer a survival advantage in patients with Ph-like ALL, particularly those with persistent MRD or adverse molecular features.

In the MD Anderson cohort, Jain et al. observed improved leukemia-free survival and reduced relapse rates in adult Ph-like ALL patients who underwent HSCT in CR1 compared to those treated with chemotherapy alone, despite the transplant group having a higher-risk disease profile at diagnosis [15]. Similarly, data from the CALGB/Alliance trials showed that AYA patients with Ph-like signatures had markedly inferior survival when treated with conventional chemotherapy without transplant [9]. In pediatric populations, the COG AALL1131 trial stratified patients based on MRD and molecular risk. Among Ph-like patients with MRD ≥ 0.01% after induction, those who received HSCT had better EFS than those who did not, although follow-up remains limited [24,26,27,29,30,31,32].

It is important to emphasize that any retrospective comparison between HSCT and non-HSCT approaches is inevitably subject to selection bias, inherent in the medical decision-making process that determines a patient’s eligibility for the procedure. This assessment takes into account several factors, such as the absence of significant medical comorbidities and the presence of adequate psychosocial and family support, which directly influence the selection of candidates.

### 5.2. Patients with MRD-Negative Status and Potential Survival Trade-Offs

The role of transplantation in MRD-negative patients with Ph-like acute lymphoblastic leukemia (ALL) remains unclear. Some studies suggest that patients who achieve a deep molecular remission may not derive significant additional benefit from hematopoietic stem cell transplantation (HSCT), particularly in the context of targeted therapies.

For example, in the COG AALL1521 study (NCT02723994), patients with CRLF2 rearrangements and/or JAK pathway mutations received ruxolitinib in combination with chemotherapy. A subgroup of these patients achieved rapid MRD clearance, raising the question of whether transplantation can be safely omitted in this context. However, long-term follow-up is needed to confirm whether durable remission can be maintained without HSCT.

It is important to emphasize that, at present, no definitive evidence confirms the long-term efficacy of this strategy, making it premature to draw firm conclusions about the impact of tyrosine kinase inhibitors (TKIs) in this population. Conversely, some data suggest the need for a more cautious approach. A notable example is the subgroup analysis presented by Dinner et al. at EHA 2025 [23], which demonstrated highly promising results in young adults with Ph-like ALL treated on the E1910 study, where the addition of blinatumomab significantly improved survival without the systematic use of HSCT.

Although transplantation may reduce relapse risk, it carries substantial risks of non-relapse mortality (NRM), graft-versus-host disease (GVHD), and long-term morbidity.

Emerging evidence advocates for a more cautious and personalized approach. The subgroup analysis by Dinner et al. at EHA 2025 [23] showed exceptionally promising outcomes in young adults with Ph-like ALL treated in the E1910 trial. In this context, adding blinatumomab to therapy resulted in a substantial survival benefit, with 3-year overall survival (OS) and relapse-free survival (RFS) rates of 100%, without the need for routine HSCT.

Further confirmation comes from the Total Therapy XV study conducted at St. Jude’s Children’s Research Hospital, where children with Ph-like ALL were treated with an intensive, risk-adapted chemotherapy regimen based on MRD assessment. This approach demonstrated that targeted treatment modulated according to molecular response can achieve excellent outcomes without generalized use of HSCT [3].

Taken together, these data suggest the need to reconsider the current therapeutic paradigm, which often views HSCT as an almost obligatory step for high-risk cases. Instead, a more sophisticated and personalized model is emerging, in which continuous MRD monitoring and integration of innovative therapies—such as immunotherapy with blinatumomab or CAR T-cell therapy—could allow for reserving transplantation only for patients who do not achieve deep molecular remission or who present with refractory disease.

This evolving scenario calls for dynamic, risk-adapted strategies rather than fixed indications for transplantation.

## 6. Future Directions in the Management of Ph-like ALL

The treatment landscape of Ph-like ALL is rapidly evolving, driven by advances in genomic profiling, targeted therapy, and cellular immunotherapy. While HSCT remains a mainstay for many high-risk patients, novel strategies may refine or even replace its role in specific clinical scenarios. The future of Ph-like ALL management lies in personalized, risk-adapted approaches based on precise molecular features and dynamic response assessment.

### 6.1. Integration of Genomic Diagnostics into Frontline Therapy

Ph-like ALL constitutes a clinically and biologically heterogeneous entity, in which cytogenetic and molecular aberrations possess significant prognostic implications. Consequently, their timely and comprehensive identification-integrated with the assessment of MRD is critical not only for accurate risk stratification but also for the optimization of therapeutic strategies. The implementation of high-resolution genomic technologies, including NGS, RNA-based fusion panels, and SNP arrays, has substantially enhanced the molecular characterization of this leukemia subtype. These platforms have facilitated the discovery of novel genetic lesions, thereby advancing our understanding of the disease’s ontogeny and clonal evolution. These techniques may potentially enable early detection of targetable kinase fusions (ABL1, ABL2, PDGFRB, JAK2); real-time inclusion of TKIs or JAK inhibitors into induction therapy; and stratification of patients for HSCT versus non-HSCT approaches based on response and genetic risk

In the future, broader implementation of molecular diagnostics may allow for treatment algorithms that replace morphology- and age-based risk models with genetically informed ones [22].

### 6.2. Targeted Therapies in Frontline Regimens

The integration of TKIs and JAK inhibitors into frontline treatment represents a promising strategy for improving outcomes in Ph-like ALL (Table 2):

- ABL-class rearrangements: these fusions (e.g., EBF1-ABL1, NUP214-ABL1) confer sensitivity to TKIs such as imatinib or dasatinib. Incorporation of TKIs into induction or consolidation therapy has shown encouraging results in case series and early-phase studies [3,27].
- CRLF2 rearrangements and JAK mutations: preclinical data and early clinical trials support the use of ruxolitinib, a JAK1/2 inhibitor, in cases harboring JAK-STAT pathway activation [25]. The COG AALL1521 trial (NCT02723994) is currently evaluating ruxolitinib in combination with chemotherapy for newly diagnosed Ph-like ALL.
- RAS pathway mutations: these may be susceptible to MEK inhibitors (e.g., trametinib), although clinical validation is still limited [19].
- The ETV6-NTRK3 fusion constitutes a putative therapeutic target for TRK inhibitors (e.g., larotrectinib); however, further rigorous clinical evaluation is necessary [29].
- The EPOR::IGH fusion has demonstrated preclinical sensitivity to combined treatment with ponatinib and ruxolitinib, warranting additional investigation [24].

Targeted therapies may not only improve remission induction but also reduce the MRD burden, potentially altering indications for transplant in selected patients. Despite promising advances, significant barriers remain. First, comprehensive genomic profiling is not uniformly available in all treatment centers, delaying or limiting timely implementation of precision therapies. Second, the efficacy of targeted agents in preventing relapse remains under investigation, and their optimal duration and combination with chemotherapy are not yet standardized. Finally, there are no prospective randomized trials specifically addressing whether incorporation of TKIs or JAK inhibitors can obviate the need for HSCT in high-risk Ph-like patients. As such, HSCT continues to be recommended in many cases, particularly when MRD persists or high-risk genomic features are present. Future trials are needed to clarify whether targeted therapies can serve as substitutes or complements to transplant in this population.

These targeted agents are being tested in ongoing trials (e.g., COG AALL1521, NCT02723994) as part of induction or post-remission therapy and may influence indications for HSCT. Cases with NTRK3 gene fusions may exhibit sensitivity to NTRK inhibitors, including larotrectinib [26]. The EPOR::IGH fusion has exhibited a synergistic therapeutic response to combined treatment with ponatinib and ruxolitinib [24].

**Table 2:** Ph-like ALL—Genetic Alterations, Potential Targeted Therapies, and Future HSCT Implications.

Genetic Alteration/Subgroup	Targetable Pathway	Candidate Therapy	Implication for HSCT
CRLF2 rearrangement ± JAK mutations	JAK-STAT	Ruxolitinib	Consider HSCT if MRD persists despite JAK inhibition
JAK1/2 mutations	JAK-STAT	Ruxolitinib	May delay HSCT if MRD-negative post-targeted therapy
ABL-class fusions (ABL1, ABL2, PDGFRB, CSF1R)	ABL tyrosine kinase	Imatinib/Dasatinib/Ponatinib	Often recommended; HSCT plus TKI maintenance
EPOR rearrangement	JAK-STAT	Ruxolitinib	HSCT considered if MRD+; ruxolitinib under study
RAS pathway mutations (KRAS, NRAS, NF1)	RAS/MAPK	MEK inhibitors (experimental)	Limited evidence; HSCT often pursued
IKZF1 deletion/IKZF1plus	Transcriptional regulation	No direct target; risk marker	High relapse risk; HSCT strongly considered
MRD ≥ 0.01% after induction	Residual disease	Blinatumomab, CAR-T, HSCT	Key criterion for HSCT recommendation

### 6.3. Targeted Therapy, Immunotherapy and CAR-T Cell Approaches

One of the key research questions is whether the use of targeted therapies can sufficiently reduce relapse risk and MRD burden to avoid HSCT in selected patients. Ongoing studies are evaluating:

- Ruxolitinib (JAK1/2 inhibitor) with chemotherapy in CRLF2- and JAK-mutated Ph-like ALL (COG AALL1521).
- Ponatinib in frontline therapy for ABL-class fusions (ALLG ALL08, NCT03571321).
- MRD-guided risk adaptation, in which transplant is deferred in patients achieving rapid molecular remission.

These trials aim to define whether targeted therapy can replace HSCT in molecular responders while reserving transplant for those with persistent MRD or unresponsive disease [30,31].

Immunotherapeutic modalities such as blinatumomab and CAR-T cell therapy are reshaping the relapse salvage paradigm.

Future research will explore whether CAR-T therapy could serve as consolidation therapy in MRD-positive CR1, particularly in patients with high transplant risk or a lack of suitable donors [9,32].

### 6.4. Role of Blinatumomab: Preliminary Evidence and Therapeutic Perspectives

Although Ph-like ALL represents a high-risk subpopulation, characterized by resistance to conventional chemotherapy and a high rate of minimal residual disease (MRD) positivity, the role of immunotherapy with blinatumomab in this specific entity has not yet been fully defined.

Currently, there are no randomized studies specifically designed to assess the efficacy of blinatumomab in Ph-like ALL. However, indirect data suggest that the addition of blinatumomab to frontline therapy could modify outcomes even in these high-risk patients.

The E1910 trial, a randomized study conducted within the ECOG-ACRIN network, demonstrated that the addition of blinatumomab to chemotherapy significantly improves OS in adult patients with B-ALL in first remission. An analysis presented at EHA 2025 [23] reported specific data on a small subgroup of young adults with BCR::ABL1-like ALL, a molecularly overlapping entity with Ph-like ALL. In this group, patients randomized to receive blinatumomab achieved 3-year OS and RFS rates of 100%, compared to 45% in the group treated with standard chemotherapy.

Although these data are preliminary and refer to a limited subgroup, they raise an important question: if a deep molecular response can be achieved with early immunotherapy, the routine indication for HSCT in first remission may not be necessary in all cases.

Additional evidence comes from the GIMEMA LAL2317 study [33], which included adult patients with Ph-like ALL treated with blinatumomab during consolidation. In this study, the drug showed significant activity in reducing MRD, even in the presence of high-risk genetic alterations (e.g., CRLF2 rearrangements, JAK mutations), with some patients proceeding to HSCT and others avoiding transplantation.

Overall, these data suggest that blinatumomab-based immunotherapy could represent a key strategy in the management of Ph-like ALL, particularly in patients who achieve an early complete molecular response. The potential to avoid allogeneic transplantation—with its well-known risks of acute toxicity and long-term complications—is especially relevant in young adults.

### 6.5. Post-HSCT Maintenance and Future Clinical Trial Designs

Post-transplant relapse remains a critical issue in Ph-like ALL. The incorporation of targeted agents or immunotherapy post-HSCT is being actively investigated:

- TKIs for patients with ABL-class fusions.
- Ruxolitinib or other JAK inhibitors for JAK-mutated subtypes.
- NTRK inhibitors for leukemic subtypes characterized by ETV6-NTRK3 gene fusions.
- MEK inhibitors for subtypes driven by aberrant RAS pathway activation.
- Blinatumomab in MRD-positive patients post-HSCT.

Randomized trials are needed to determine the optimal agent, timing, and duration of maintenance therapy to prevent relapse and prolong survival.

The future of Ph-like ALL management will depend on well-designed, biologically stratified clinical trials. Important features for future studies include:

- Early molecular diagnosis and enrollment.
- MRD-based risk adaptation.
- Randomization between HSCT and non-HSCT strategies in molecular responders.
- Evaluation of combinations of targeted therapy + immunotherapy.

Furthermore, ensuring global access to genomic testing and novel agents is essential for equitable implementation of precision medicine in Ph-like ALL, especially in low- and middle-income countries.

## 7. Conclusions

Ph-like ALL represents a high-risk molecular subtype of B-ALL characterized by adverse alterations. MRD clearance is generally suboptimal post induction therapy and correlates with inferior relapse-free survival outcomes. HSCT has long been considered a cornerstone in the treatment of high-risk ALL and remains a critical option for many patients with Ph-like ALL—particularly those with persistent MRD or unfavorable genetic features. However, the landscape of Ph-like ALL management is rapidly evolving. Advances in genomic diagnostics now enable early identification of targetable lesions, allowing for the timely integration of TKIs, JAK inhibitors, and other precision therapies into frontline regimens. Preliminary data suggest that these targeted approaches may enhance MRD clearance and improve outcomes, potentially reducing the need for transplant in selected patients.

Retrospective studies support the role of HSCT in CR1 for high-risk patients, but the lack of prospective, randomized data specifically focused on Ph-like ALL limits our ability to define standardized transplant indications. The development of immunotherapeutic options, such as blinatumomab and CAR-T cell therapy, offers additional tools to eradicate disease pre- or post-transplant, and may further shift the role of HSCT in this setting. Ultimately, the future of Ph-like ALL treatment lies in individualized, risk-adapted strategies that integrate genomic profiling, dynamic MRD assessment, and access to novel therapies. Prospective trials are urgently needed to define the optimal use of HSCT versus non-HSCT approaches and to determine how best to combine transplant with targeted and immunotherapeutic interventions. Until then, multidisciplinary decision-making remains essential to guide treatment for this challenging leukemia subtype.
